# Consumption of regular-fat vs reduced-fat cheese reveals gender-specific changes in LDL particle size - a randomized controlled trial

**DOI:** 10.1186/s12986-018-0300-0

**Published:** 2018-09-21

**Authors:** Farinaz Raziani, Parvaneh Ebrahimi, Søren Balling Engelsen, Arne Astrup, Anne Raben, Tine Tholstrup

**Affiliations:** 1 Department of Nutrition, Exercise and Sports, Faculty of Science, University of Copenhagen, Rolighedsvej 26, 1958 Frederiksberg C, Denmark; 2 Department of Food Science, Faculty of Science, University of Copenhagen, Rolighedsvej 26, 1958 Frederiksberg C, Denmark

**Keywords:** Saturated fat, NMR, Dairy, Metabolic syndrome

## Abstract

**Background:**

Regular-fat cheese does not seem to increase low density lipoprotein cholesterol (LDL-C) concentrations compared to reduced-fat cheese. However, plasma LDL-C concentrations do not reflect levels and size of LDL particles, which might be a better predictor of cardiovascular risk.

**Methods:**

The aim was to compare the effects of regular-fat cheese vs reduced-fat cheese and carbohydrate-rich foods on LDL particle size distribution in adults with ≥ 2 metabolic syndrome (MetS) risk factors. The study was part of a 12 weeks’ randomized controlled trial in which subjects had been randomly allocated to 1 of 3 intervention groups; regular-fat cheese (REG), reduced-fat cheese (RED) or a no-cheese/carbohydrate (CHO) group. Subjects in the REG and RED groups consumed 80 g cheese/d per 10 MJ, whereas subjects in the CHO consumed bread and jam corresponding to 90 g/d and 25 g/d per 10 MJ, respectively. Fasting blood samples at wk. 0 (baseline) and wk. 12 were analyzed for LDL particle size distribution and cholesterol content using nuclear magnetic resonance (NMR) spectroscopy.

**Results:**

A total of 85 subjects [mean ± SD age: 54.0 ± 12.8 y; BMI: 28.7 ± 3.6 kg/m^2^] completed the study. Overall, regular-fat cheese did not impact lipoprotein particle number and size differently than reduced-fat cheese. In men (*n* = 23), the REG diet decreased total LDL particle number (LDL-P, − 223.2 ± 91.1 nmol/l, *P* = 0.01) compared with the RED diet. The reduction was primarily in the medium-sized LDL fraction (− 128.5 ± 51.8 nmol/l, *P* = 0.01). In women (*n* = 62), the REG diet increased the concentration of cholesterol in the small high density lipoprotein (HDL) particles compared with the CHO diet (2.9 ± 1.0 mg/dl, *P* = 0.006).

**Conclusion:**

Overall, regular-fat cheese did not alter LDL particle size distribution compared to reduced-fat cheese after a 12 wk. intervention in subjects with ≥2 MetS risk factors. However, our results suggest that lipoprotein response to cheese intake is gender-specific. This warrants further investigation.

**Trial registration:**

This trial was registered at Clinicaltrials.gov as NCT0261471. Registered 30 November 2015 - Retrospectively registered.

## Background

Epidemiological studies have revealed that reducing plasma cholesterol levels results in clinically relevant reductions in risk factors for cardiovascular disease (CVD) [1]. Although increased plasma low density lipoprotein cholesterol (LDL-C) concentrations is considered one of the main CVD risk factors, a number of individuals who develop atherosclerosis and CVD have LDL-C concentrations in the normal range [2]. The main reason for this observation is that LDL particles are heterogeneous in respect to size, density, and physiochemical properties [3]. In the case of LDL, the concentration of LDL-C does not reflect the levels of LDL particle size. Studies show that a higher presence of small, dense LDL (sdLDL) particles is associated with a higher risk of CVD [4, 5], independent of plasma LDL-C concentrations [6, 7].

The main focus of dietary recommendations for CVD prevention and management has been a reduction of dietary fat, in particular saturated fatty acids [8]. Interestingly, Dreon et al. found a positive correlation between SFA intake and large, buoyant LDL particles, with no such association with poly- or mono-unsaturated fatty acids [9]. Another study found that substituting saturated fat with carbohydrates resulted in smaller and denser LDL particles [10]. Thus, the benefit of reducing the intake of foods with high amounts of saturated fat is highly dependent on what is eaten instead.

Cheese is a dairy product with a high content of saturated fat. Therefore, it is a common perception that cheese increases plasma LDL-C and in turn CVD risk. However, human intervention studies show that cheese has a neutral effect on plasma cholesterol levels when compared with butter intake [11]. Likewise, prospective studies found no association between cheese intake and CVD risk [12, 13] or increased risk of all-cause mortality [14]. One of the explanations behind the findings from prospective studies might be that saturated fat in cheese results in larger LDL particle size. This has, however not yet been confirmed in a clinical trial.

The primary objective of the current study was to compare the effect of cheese intake with different fat content and an iso-caloric amount of carbohydrate-rich foods on LDL particle size distribution in a metabolically vulnerable study population. Based on studies showing that the increase in LDL-C induced by saturated fat results in a higher proportion of larger and less atherogenic LDL particles [15–18], we hypothesized that intake of regular-fat cheese would be associated with larger LDL particles compared to reduced-fat cheese and a carbohydrate-rich control diet.

## Methods

### Study design

The present study was part of a larger study, which has been described in detail elsewhere [19]. In brief, 164 subjects (18–70 years of age) were recruited for a 12-wk randomized, parallel intervention, with a 2-wk run-in period. Subjects were allocated to three intervention groups; regular-fat cheese (REG), reduced-fat cheese (RED), or non-cheese/carbohydrate (CHO). Fasting blood samples were drawn after the 2-wk run-in period at baseline (wk 0) and after the 12-wk intervention period. The study was carried out at the Department of Nutrition, Exercise and Sports, Faculty of Sciences, University of Copenhagen, Frederiksberg, Denmark, from February 2014 to May 2015 and was approved by the Municipal Ethical Committee of Copenhagen (H-4-2013-099). All of the subjects gave their informed consent in writing after receiving written and oral information about the study.

### Subjects

In this sub-study, EDTA plasma was collected at wk. 0 and wk. 12 from the 164 subjects included in the main intervention. However, due to technical issues, only blood samples collected from 85 subjects were stored properly and could be used for analysis in this sub-study.

Inclusion criteria were 18–70 y of age, BMI (in kg/m^2^) of 18.5–37.5, waist circumference > 80 cm for women and > 94 cm for men, and ≥ 1 additional established risk factor for the MetS; systolic blood pressure > 130 mmHg and/or diastolic blood pressure > 85 mmHg, triacylglycerols > 1.7 mmol/l, HDL cholesterol < 1.00 mmol/l for men and < 1.3 mmol/l for women, and/or glucose > 5.6 mmol/l. Exclusion criteria were chronic diseases (known T2D, CVD, or other chronic diseases that could affect the study outcome), milk allergy, the use of prescription medicine that could affect the results of the study (e.g., lipid-lowering agents), 10 h of strenuous physical activity/wk., drug and alcohol abuse, blooddonation,1 mo before study commencement or during the study period, simultaneous participation in other clinical studies, pregnant or lactating woman or women who were planning to become pregnant during the intervention, or inability to comply with the procedures required by the protocol.

Subjects (62 women and 23 men) with ≥2 metabolic syndrome risk factors had a mean BMI of 28.7 ± 3.6 kg/m^2^ and a mean age of 54.0 ± 12.8 y. Baseline characteristics of the 85 subjects included in this study are listed in Table 1.

**Table 1:** Subject baseline characteristics^a^

	REG (*n* = 30)	RED (*n* = 26)	CHO (*n* = 29)
Sex [*n* (%)]			
Women	21(70)	21 (81)	20 (69)
Men	9 (30)	5 (19)	9 (31)
Age (y)	54.8 ± 13.7	51.8 ± 13.4	55.0 ± 11.6
Weight (kg)	84.2 ± 14.3	84.5 ± 11.2	84.3 ± 16.5
BMI (kg/m^2^)	29.3 ± 3.6	28.4 ± 3.0	28.4 ± 4.0
Waist circumference (cm)	98.1 ± 11.6	96.7 ± 10.3	96.9 ± 10.9
Smoking [n (%)]	1 (3)	2 (7)	1 (3)
Energy requirement (MJ/d)	10.3 ± 1.9	10.3 ± 1.8	10.2
Systolic blood pressure (mmHG)	133.2 ± 16.3	129.7 ± 14.1	128.0 ± 16.8
Diastolic blood pressure (mmHG)	85.7 ± 9.1	82.8 ± 8.6	84.0 ± 8.3

^a^Values are means ± SDs unless otherwise indicated. *n* = 85. *CHO* carbohydrate control, *RED* reduced-fat cheese, *REG* regular-fat cheese

### Experimental diets

The energy content and macronutrient distribution of the three test foods have been described in detail elsewhere [19]. In short, subjects in the REG and RED groups were provided with equal amounts of regular-fat Danbo (Riberhus, 25% fat, Arla, Denmark) and cheddar (Sharp Cheddar, 32% fat, Lactalis, Scotland), and reduced-fat Danbo (Riberhus,13% fat, Arla, Denmark) and cheddar (Sharp Cheddar, 16% fat, Lactalis, Scotland), respectively. In the CHO group, cheese was replaced with white wheat bread (Kohberg, Denmark) and sugar-sweetened jam (Fynbo, Denmark). Subjects in all three groups were also provided with 250 ml of skimmed milk (0.1% fat, Arla, Denmark) per day throughout the intervention. No other dairy products were allowed during the intervention. The daily amounts of cheese provided corresponded to 80 g/d per 10 MJ, while the daily amounts of bread and jam corresponding to 90 g/d and 25 g/d per 10 MJ, respectively.

### Dietary records

In order to provide information about dietary intake during the intervention, subjects completed a 3-d dietary food records at wk. 0 and wk. 12. Two week-days and one weekend day were included in the dietary record to take any differences in nutrient intake during weekdays and weekend days into account.

## Analytic procedures

### ^1^H NMR lipoprotein subclass analysis

Serum samples, stored in EDTA containing tubes, were prepared and measured according to Bruker’s sample preparation standard operating procedures (SOPs) and In Vitro Diagnostics research (IVDr) platform, which allow highly standardized and reproducible NMR measurements of biofluids, as reported previously [20]. The spectrometer was a Bruker Avance-III 600 spectrometer (Bruker Biospin Gmbh, Rheinstetten, Germany) operating at a proton frequency of 600.13 MHz (14.1 T), equipped with a double tuned broadband inverse (BBI) probe for 5-mm sample tubes. Bruker IVDr Lipoprotein Subclass Analysis (B.I.-LISA) was then used to analyze the resulted ^1^H NMR spectra and calculate lipoproteins subclasses. In brief, this platform uses a regression model that is developed using ultracentrifugation results of a training dataset, which allows for prediction of the concentrations of the lipoproteins from the ^1^H NMR spectra of new plasma samples without any further need for ultra-centrifugation [21, 22]. This model provides information on main lipoprotein classes, including very low density lipoprotein (VLDL), intermediate density lipoprotein (IDL), LDL, and high density lipoprotein (HDL), as well as the six VLDL subclasses (VLDL-1 to VLDL-6), six LDL subclasses (LDL-1 to LDL-6), and four HDL subclasses (HDL-1 to HDL-4). Subclasses were sorted according to their increasing density and decreasing size in ascending order, respectively. Compositional information of main –and subclasses consists of lipids, i.e., cholesterol, free cholesterol, triglycerides, phospholipids and apolipoproteins; Apo-A1, Apo-A2 and Apo-B in mg/dl unit. Moreover, information on particle numbers for VLDL, IDL and LDL main classes, and VLDL and LDL subclasses were also obtained from B.I.-LISA, in nmol/L unit.

### Statistical analysis

All statistical analyses were performed using R (R Core Team, 2015) [23]. Linear mixed models were used to evaluate the effect of the intervention on all outcomes. Baseline outcome values were included as covariates. Adjustments for age, sex, BMI, and changes in body fat percent (from wk. 0 to wk. 12) were also included as fixed effects. As a significant effect of sex was observed, separate sub-analysis for men and women were carried out for all outcomes. Differences between periods were captured by means of random effects. For each outcome two pairwise comparisons were evaluated: REG versus RED and REG versus CHO, using post hoc t-test based on the linear mixed models; *p*-values were adjusted for multiplicity [24]. Model checking of assumptions of homogeneity of variance and normal distribution was carried out by means of residual and normal probability plots. Results are presented as means ± SEMs. A significance level of 0.05 was used.

## Results

### Anthropometrics

Changes in mean anthropometric measures, body composition, and blood pressure are shown in Table 2. There were no significant differences in body weight, fat mass, lean body mass, waist circumference, or blood pressure between REG and RED diets or between the REG and CHO diets (all *P* ≥ 0.05).

**Table 2:** Fasting values of anthropometric measurements, body composition and blood pressure at week 12 and changes from baseline^a^

	REG (*n* = 30)		RED (*n* = 26)		CHO (*n* = 29)		REG vs. RED	REG vs. CHO
	Week 12	Change from baseline	Week 12	Change from baseline	Week 12	Change from baseline	*P*	*P*
Body weight (kg)	84.6 ± 2.7	0.4 ± 0.3	85.1 ± 2.4	0.6 ± 0.3	84.5 ± 3.3	0.2 ± 0.3	0.39	0.91
WC (cm)	98.7 ± 2.0	0.5 ± 0.4	98.4 ± 2.0	1.1 ± 0.5	96.9 ± 2.3	0.0 ± 0.6	0.52	0.46
BMI (kg/m^2^)	29.5 ± 0.7	0.1 ± 0.1	28.8 ± 0.6	0.2 ± 0.1	28.5 ± 0.8	0.1 ± 0.1	0.61	0.89
Fat Mass (kg)	32.2 ± 1.6	0.0 ± 0.2	34.6 ± 1.4	0.7 ± 0.3	32.1 ± 1.9	− 0.3 ± 0.3	0.09	0.71
Fat percent (%)	38.1 ± 1.3	− 0.2 ± 0.2	40.6 ± 1.2	0.4 ± 0.3	38.0 ± 1.6	− 0.2 ± 0.3	0.17	0.88
Lean Body Mass (kg)	49.2 ± 1.9	0.3 ± 0.2	47.2 ± 1.7	0.0 ± 0.3	48.9 ± 2.3	0.1 ± 0.3	0.65	0.54
Systolic BP (mmHg)	132.3 ± 2.5	− 1.0 ± 2.0	126.0 ± 2.0	−4.0 ± 1.7	126.6 ± 2.4	− 1.4 ± 2.2	0.09	0.14
Diastolic BP (mmHg)	83.9 ± 1.6	− 1.8 ± 0.9	80.4 ± 1.7	− 2.3 ± 1.1	81.7 ± 1.3	− 2.2 ± 1.0	0.35	0.48

^a^All values are means ± SEMs. Significant differences between groups are based on linear mixed models with baseline values as covariates and adjustments for age, sex, BMI, and change in body fat. Pairwise comparisons were made by using a post hoc t test on the linear mixed model with *P* values adjusted for multiplicity. *BP* blood pressure, *CHO* carbohydrate control, *RED* reduced-fat cheese, *REG* regular-fat cheese, *WC* waist circumference

### Lipoprotein particle number

Changes from baseline as well as differences between groups for total VLDL-P and its subclasses, IDL-P, and LDL-P and its subclasses are shown in Table 3. Overall, there were no significant differences for these outcomes between the REG diet and RED diet or between REG and CHO diet (all *P* ≥ 0.16). The gender sub analysis showed similar results in women (all *P* ≥ 0.17), (Table 4). However, the gender sub analysis showed a decrease in LDL-P in men on the REG diet compared to the RED diet (− 223.2 ± 91.1 nmol/l, *P* = 0.01) (Table 5). The decrease in LDL-P in men was primarily due to a lower number of medium-sized LDL particles (− 128.5 ± 51.8 nmol/l, *P* = 0.01). Ratios of LDL-C:HDL-C and ApoB:ApoA-1 were significantly reduced in men on the REG diet compared with the RED diet (− 0.53 ± 0.13, *P* = 0.0007 and 0.09 ± 0.03, *P* = 0.009, respectively), but did not differ from that of the CHO diet (Table 5).

**Table 3:** Lipoprotein particle number and cholesterol content at wk. 12 and changes from baseline in men and women^a^

	REG (*n* = 30)		RED (*n* = 26)		CHO (*n* = 29)		REG vs. RED	REG vs. CHO
	Week 12	Change from baseline	Week 12	Change from baseline	Week 12	Change from baseline	*P*	*P*
VLDL-P (nmol/l)^2^	148.4 ± 14.1	− 3.6 ± 15.3	129.5 ± 9.4	−6.0 ± 8.8	126.1 ± 13.8	3.4 ± 6.7	0.56	0.67
IDL-P (nmol/L)	73.3 ± 6.9	− 4.5 ± 6.9	68.5 ± 6.5	− 6.1 ± 5.0	68.2 ± 7.0	2.1 ± 4.3	0.62	0.81
LDL-P (nmol/l)								
Total	1322 ± 60.0	26.2 ± 41.3	1265 ± 78.8	7.9 ± 50.2	1363 ± 55.5	− 2.9 ± 39.1	0.43	0.99
LDL_1 + 2_^b^	415.9 ± 21.4	22.2 ± 15.1	444.2 ± 23.0	− 13.3 ± 19.3	453.2 ± 22.4	−29.3 ± 14.7	0.82	0.16
LDL_3 + 4_^b^	403.2 ± 20.6	9.9 ± 22.4	414.9 ± 31.6	15.9 ± 19.5	403.3 ± 22.2	− 10.8 ± 18.1	0.98	0.76
LDL_5 + 6_^b^	487.7 ± 43.1	− 12.7 ± 20.5	391.1 ± 43.6	−7.7 ± 25.7	479.6 ± 49.3	32.0 ± 19.0	0.39	0.35
VLDL-C (mg/dl)								
Total^b^	16.5 ± 2.2	−0.5 ± 2.4	13.2 ± 1.6	−1.3 ± 1.3	12.8 ± 2.4	0.7 ± 1.2	0.47	0.68
VLDL_1 + 2_	7.3 ± 1.2	0.7 ± 1.1	4.9 ± 0.9	− 0.5 ± 0.6	5.0 ± 1.3	0.1 ± 0.1	0.26	0.56
VLDL_3 + 4_^b^	6.7 ± 1.0	− 1.2 ± 1.3	5.6 ± 0.7	−1.0 ± 0.7	5.4 ± 1.0	− 0.2 ± 0.5	0.67	0.87
VLDL_5 + 6_^b^	2.1 ± 0.1	− 0.1 ± 0.1	2.0 ± 0.1	0.0 ± 0.1	2.0 ± 0.1	0.7 ± 0.7	0.96	0.68
IDL-C (mg/dl)	7.0 ± 1.1	− 0.2 ± 0.9	6.7 ± 1.1	−1.5 ± 1.3	7.1 ± 1.3	0.1 ± 0.7	0.28	0.81
LDL-C (mg/dl)								
Total	118.2 ± 5.4	3.7 ± 4.4	117.3 ± 7.3	3.8 ± 4.9	122.6 ± 4.5	−2.6 ± 3.9	0.69	0.70
LDL_1 + 2_^b^	42.8 ± 2.5	2.7 ± 1.7	46.6 ± 2.6	−0.5 ± 2.3	47.2 ± 2.4	−3.2 ± 1.9	0.51	0.19
LDL_3 + 4_^b^	38.2 ± 2.2	0.9 ± 2.1	40.0 ± 3.1	2.3 ± 2.1	38.7 ± 2.2	−1.7 ± 1.8	0.72	0.57
LDL_5 + 6_^b^	36.2 ± 3.1	− 0.5 ± 1.4	29.6 ± 3.4	0.2 ± 2.0	35.7 ± 3.5	2.1 ± 1.5	0.88	0.21
HDL-C (mg/dl)								
Total	56.0 ± 2.9	2.2 ± 1.0	56.5 ± 2.5	2.4 ± 1.3	55.9 ± 2.3	− 0.6 ± 1.3	0.84	0.03
HDL_1 + 2_^b^	27.1 ± 2.7	1.0 ± 0.8	27.5 ± 2.1	0.6 ± 1.0	28.6 ± 2.0	0.1 ± 1.1	0.68	0.23
HDL_3 + 4_^b^	29.4 ± 0.8	1.2 ± 0.6	29.4 ± 0.9	1.6 ± 0.7	27.8 ± 0.9	− 0.8 ± 0.6	0.95	0.02
LDL-C:HDL-C ratio	2.2 ± 0.1	0.0 ± 0.1	2.2 ± 0.2	0.0 ± 0.1	2.3 ± 0.1	0.0 ± 0.1	0.62	0.81
Apo-B:Apo-A1 ratio	0.6 ± 0.0	0.0 ± 0.0	0.6 ± 0.0	0.0 ± 0.1	0.7 ± 0.0	0.0 ± 0.0	0.67	0.16

^a^All values are means ± SEMs. Statistical differences between groups are based on linear mixed models with baseline values as covariates and adjustments for age, sex, BMI and change in body fat. Pairwise comparisons were made using post hoc t-test on the linear mixed model with *p*-values adjusted for multiplicity. ^b^Significant sex effect observed. Abbreviations: *Apo* apolipoprotein, *CHO* carbohydrate control, *HDL-C* high density lipoprotein cholesterol, *IDL-C* intermediate density lipoprotein cholesterol, *IDL-P* intermediate density lipoprotein particle number, *LDL-C* low density lipoprotein cholesterol, *LDL-P* low density lipoprotein particle number, *RED* reduced-fat cheese, *REG* regular-fat cheese, *VLDL-C* very low density lipoprotein cholesterol, *VLDL-P* very low density lipoprotein particle number

**Table 4:** Lipoprotein particle number and cholesterol content at wk. 12 and changes from baseline in women^a^

	RED (*n* = 21)		RED (*n* = 21)		CHO (*n* = 20)		REG vs. RED	REG vs. CHO
	Week 12	Change from baseline	Week 12	Change from baseline	Week 12	Change from baseline	*P*	*P*
VLDL-P (nmol/l)	135.6 ± 13.5	− 11.2 ± 20.0	126.7 ± 10.6	− 10.4 ± 10.3	104.4 ± 13.3	− 6.4 ± 7.0	0.59	0.25
IDL-P (nmol/L)	68.8 ± 6.1	−4.9 ± 9.3	68.5 ± 6.8	− 7.6 ± 5.7	63.7 ± 7.2	1.3 ± 5.5	0.75	0.91
LDL-P (nmol/l)								
Total	1343.0 ± 57.4	72.4 ± 53.1	1238.3 ± 81.0	− 30.9 ± 49.4	1359.7 ± 71.9	11.3 ± 52.7	0.08	0.83
LDL_1 + 2_	449.9 ± 24.1	31.0 ± 19.6	459.9 ± 25.0	−14.2 ± 23.0	497.4 ± 23.5	− 22.3 ± 21.0	0.41	0.68
LDL_3 + 4_	471.6 ± 19.2	45.2 ± 25.5	408.4 ± 35.1	− 0.5 ± 17.7	422.8 ± 26.2	1.2 ± 24.1	0.24	0.51
LDL_5 + 6_	451.8 ± 42.6	−9.1 ± 25.9	354.5 ± 37.7	31.4 ± 23.6	407.7 ± 47.3	25.2 ± 18.8	0.17	0.25
VLDL-C (mg/dl)								
Total	13.8 ± 2.0	−1.9 ± 3.0	12.7 ± 1.8	−2.0 ± 1.6	9.1 ± 2.1	− 0.9 ± 1.2	0.65	0.30
VLDL_1 + 2_	5.8 ± 1.2	0.1 ± 1.2	4.5 ± 1.0	− 0.8 ± 0.8	2.9 ± 1.1	−0.4 ± 0.6	0.31	0.21
VLDL_3 + 4_	5.6 ± 0.8	− 1.8 ± 1.7	5.6 ± 0.9	− 1.3 ± 0.9	4.1 ± 0.9	− 0.7 ± 0.5	0.94	0.51
VLDL_5 + 6_	2.0 ± 0.1	− 0.1 ± 0.2	2.1 ± 0.1	0.0 ± 0.1	1.8 ± 0.1	0.0 ± 0.1	0.62	0.83
IDL-C (mg/dl)	5.9 ± 0.9	−0.6 ± 1.1	7.3 ± 1.3	− 1.6 ± 1.6	6.1 ± 1.2	− 0.2 ± 0.8	0.83	0.60
LDL-C (mg/dl)								
Total	121.3 ± 5.7	8.3 ± 5.5	114.9 ± 7.9	0.4 ± 4.8	124.6 ± 6.0	−0.3 ± 5.2	0.21	0.79
LDL_1 + 2_	46.4 ± 2.9	3.4 ± 2.2	48.2 ± 2.8	−0.6 ± 2.7	51.9 ± 2.5	−2.3 ± 2.2	0.60	0.74
LDL_3 + 4_	40.0 ± 2.2	3.9 ± 2.3	39.4 ± 3.4	0.8 ± 1.9	40.9 ± 2.6	− 0.4 ± 2.4	0.37	0.53
LDL_5 + 6_	33.7 ± 3.2	−0.2 ± 1.7	26.6 ± 3.0	−1.7 ± 1.7	30.7 ± 3.5	2.0 ± 1.5	0.19	0.29
HDL-C (mg/dl)								
Total	61.2 ± 3.2	2.8 ± 1.2	57.9 ± 2.8	2.7 ± 1.4	59.2 ± 2.8	−1.3 ± 1.8	0.73	0.02
HDL_1 + 2_	31.7 ± 3.1	1.1 ± 1.0	28.9 ± 2.4	0.9 ± 1.1	32.1 ± 2.4	−0.3 ± 1.5	0.77	0.21
HDL_3 + 4_	30.1 ± 1.0	1.6 ± 0.8	29.3 ± 1.0	1.6 ± 0.7	27.7 ± 1.0	−1.2 ± 0.7	0.63	0.006
LDL-C:HDL-C ratio	2.1 ± 0.1	0.1 ± 0.1	2.1 ± 0.2	−0.1 ± 0.1	2.2 ± 0.1	0.1 ± 0.1	0.20	0.69
Apo-B:Apo-A1 ratio	0.6 ± 0.0	0.0 ± 0.0	0.6 ± 0.0	0.0 ± 0.0	0.6 ± 0.0	0.0 ± 0.0	0.23	0.19

^a^All values are means ± SEMs. Statistical differences between groups are based on linear mixed models with baseline values as covariates and adjustments for age, BMI and change in body fat. Pairwise comparisons were made using post hoc t-test on the linear mixed model with p-values adjusted for multiplicity. Abbreviations: *Apo* apolipoprotein, *CHO* carbohydrate control, *HDL-C* high density lipoprotein cholesterol, *IDL-C* intermediate density lipoprotein cholesterol, *IDL-P* intermediate density lipoprotein particle number, *LDL-C* low density lipoprotein cholesterol, *LDL-P* low density lipoprotein particle number, *RED* reduced-fat cheese, *REG* regular-fat cheese, *VLDL-C* very low density lipoprotein cholesterol, *VLDL-P* very low density lipoprotein particle number

**Table 5:** Lipoprotein particle number and cholesterol content at wk. 12 and changes from baseline in men^a^

	REG (*n* = 9)		RED (*n* = 5)		CHO (*n* = 9)		REG vs. RED	REG vs. CHO
	Week 12	Change from baseline	Week 12	Change from baseline	Week 12	Change from baseline	*P*	*P*
VLDL-P (nmol/l)	178.3 ± 34.0	14.3 ± 21.1	141.3 ± 21.7	12.5 ± 14.8	174.5 ± 28.2	25.2 ± 13.0	0.56	0.12
IDL-P (nmol/L)	83.9 ± 18.5	−3.4 ± 8.2	68.2 ± 19.7	0.1 ± 10.9	78.0 ± 16.0	3.8 ± 7.2	0.61	0.24
LDL-P (nmol/l)								
Total	1275.3 ± 154.4	−81.7 ± 45.5	1381.7 ± 244.9	171.0 ± 150.1	1372.0 ± 86.3	−34.6 ± 48.5	0.01	0.68
LDL_1 + 2_	336.4 ± 31.8	1.6 ± 21.3	378.1 ± 51.9	−9.6 ± 31.3	355.0 ± 31.3	−44.8 ± 8.6	0.45	0.05
LDL_3 + 4_	369.5 ± 53.0	−72.4 ± 32.2	441.8 ± 79.5	84.5 ± 65.9	359.9 ± 40.3	−37.3 ± 21.8	0.01	0.53
LDL_5 + 6_	571.4 ± 103.3	−21.1 ± 33.9	544.8 ± 156.7	91.8 ± 81.7	639.2 ± 104.8	47.1 ± 46.2	0.06	0.12
VLDL-C (mg/dl)								
Total	22.7 ± 5.4	2.7 ± 4.1	15.1 ± 3.4	1.5 ± 1.7	21.0 ± 5.1	4.1 ± 2.7	0.97	0.22
VLDL_1 + 2_	10.6 ± 2.6	1.9 ± 2.5	6.7 ± 2.1	0.9 ± 1.0	9.6 ± 2.9	3.0 ± 1.6	0.79	0.28
VLDL_3 + 4_	9.3 ± 2.7	0.3 ± 1.6	5.7 ± 1.4	0.4 ± 1.4	8.3 ± 2.4	1.1 ± 1.2	0.67	0.19
VLDL_5 + 6_	2.2 ± 0.4	0.1 ± 0.2	1.8 ± 0.1	0.0 ± 0.3	2.4 ± 0.3	0.1 ± 0.2	0.76	0.72
IDL-C (mg/dl)	9.6 ± 3.0	0.7 ± 1.6	4.3 ± 1.5	−1.3 ± 2.3	9.4 ± 3.2	0.8 ± 1.5	0.69	0.55
LDL-C (mg/dl)								
Total	111.0 ± 12.8	−6.9 ± 5.8	127.1 ± 20.1	18.2 ± 15.7	118.3 ± 6.1	−7.8 ± 5.1	0.04	0.82
LDL_1 + 2_	34.3 ± 3.5	1.0 ± 2.5	40.1 ± 6.0	0.0 ± 4.0	36.8 ± 3.5	−5.1 ± 1.2	0.88	0.03
LDL_3 + 4_	33.9 ± 5.1	−6.2 ± 3.5	42.5 ± 7.5	8.4 ± 7.2	33.7 ± 4.1	−4.5 ± 2.3	0.03	0.76
LDL_5 + 6_	42.1 ± 7.3	−1.2 ± 2.3	42.1 ± 11.7	8.6 ± 6.8	47.0 ± 7.0	2.6 ± 3.4	0.04	0.29
HDL-C (mg/dl)								
Total	43.8 ± 3.7	1.0 ± 2.0	50.8 ± 5.4	1.0 ± 3.0	48.5 ± 2.5	1.0 ± 1.3	0.72	0.88
HDL_1 + 2_	16.4 ± 3.4	0.6 ± 1.4	21.6 ± 3.2	−0.8 ± 1.7	20.8 ± 2.4	1.0 ± 1.0	0.09	0.49
HDL_3 + 4_	27.7 ± 1.7	0.4 ± 0.8	29.7 ± 2.3	1.6 ± 1.8	28.1 ± 1.8	0.1 ± 1.0	0.36	0.73
LDL-C:HDL-C ratio	2.7 ± 0.3	−0.2 ± 0.1	2.6 ± 0.4	0.3 ± 0.2	2.5 ± 0.2	−0.2 ± 0.1	0.0007	0.72
ApoB:ApoA1 ratio	0.7 ± 0.1	−0.1 ± 0.0	0.7 ± 0.1	0.0 ± 0.0	0.7 ± 0.1	0.0 ± 0.0	0.009	0.96

^a^All values are means ± SEMs. Statistical differences between groups are based on linear mixed models with baseline values as covariates and adjustments for age, BMI and change in body fat. Pairwise comparisons were made using post hoc t-test on the linear mixed model with *p*-values adjusted for multiplicity. Abbreviations: *Apo* apolipoprotein, *CHO* carbohydrate control, *HDL-C* high density lipoprotein cholesterol, *IDL-C* intermediate density lipoprotein cholesterol, *IDL-P* intermediate density lipoprotein particle number, *LDL-C* low density lipoprotein cholesterol, *LDL-P* low density lipoprotein particle number, *RED* reduced-fat cheese, *REG* regular-fat cheese, *VLDL-C* very low density lipoprotein cholesterol, *VLDL-P* very low density lipoprotein particle number

### Cholesterol concentration in lipoprotein subclasses

Total VLDL-C and LDL-C did not differ between the diets (all *P* ≥ 0.2) (Table 3). However, the gender sub analysis in men showed a reduction of total LDL-C (− 19.4 ± 9.5 mg/dl, *P* = 0.04). The decrease in total LDL-C in men was due to a decreased concentration of cholesterol in the medium-sized (LDL_3 + 4_) and small-sized (LDL_5 + 6_) LDL particles in the REG diet compared to the RED diet (− 11.3 ± 5.2 mg/dl, *P* = 0.03 and − 10.1 ± 5.0 mg/dl, *P* = 0.04, respectively) (Table 5). Total HDL-C was higher on the REG diet compared with the CHO diet (3.5 ± 1.6 mg/dl, *P* = 0.03) (Table 3). The increase in total HDL-C was due to a significantly higher concentration of cholesterol in the smaller HDL particles (HDL_3 + 4_) on the REG diet compared with the CHO diet (1.9 ± 0.8 mg/dl, *P* = 0.02). The gender sub analysis in women showed similar results with an increase in total HDL-C and cholesterol in the smaller HDL particles (HDL_3 + 4)_ on the REG diet compared to the CHO diet (4.8 ± 2.1 mg/dl, *P* = 0.02 and 2.9 ± 1.0 mg/dl, *P* = 0.006, respectively) (Table 4). In contrast, the gender sub analysis in men showed no differences in HDL-C between the diets (Table 5).

### Dietary records

Results from the dietary records are shown in Table 6. Energy intake did not differ between groups, although subjects in the REG group tended to have a higher energy intake compared with the CHO group (*P* = 0.08). Fat intake (in g and percentage of energy) was higher during the REG intervention than during the CHO (*P* < 0.001) diets, but did not differ between the REG and RED diets. There was a higher intake of saturated fat during the REG diet compared to the RED and CHO diets (*P* < 0.01 and *P* < 0.001, respectively). The amount of MUFAs was significantly higher with the REG diet than with the CHO diet (*P* < 0.01). As expected, the intake of carbohydrate was greater with the CHO diet than with the REG diet (*P* < 0.001).

**Table 6:** Average daily consumption of energy and macronutrients for the REG, RED and CHO diet during the 12 weeks intervention^a^

	REG (*n* = 27)	RED (*n* = 25)	CHO (*n* = 27)	*P*_REGvs.RED_	*P*_REGvs.CHO_
Total energy (kJ)	9150 ± 356	8593 ± 557	7973 ± 437	0.56	0.08
Energy density (kcal/g)	1.0 ± 0.1	0.9 ± 0.1	0.9 ± 0.1	0.44	0.70
Fat (% of energy)	36.2 ± 1.3	33.5 ± 0.8	30.0 ± 1.3	0.12	< 0.001
Fat (g)	90.7 ± 5.6	77.5 ± 5.5	65.6 ± 4.6	0.11	< 0.001
Saturated fat (g)	33.3 ± 1.7	27.2 ± 1.7	19.0 ± 1.4	< 0.01	< 0.001
Monounsaturated fat (g)	31.0 ± 2.5	27.1 ± 2.2	24.6 ± 2.1	0.21	< 0.01
Polyunsaturated fat (g)	13.3 ± 1.2	12.7 ± 1.1	13.8 ± 1.2	0.71	0.99
Carbohydrate (% of energy)	40.2 ± 1.6	43.0 ± 1.1	49.7 ± 1.6	0.20	< 0.001
Dietary Fiber (g/d)	23.9 ± 1.3	22.0 ± 1.5	23.4 ± 2.6	0.44	0.85
Protein (% of energy)	19.4 ± 0.8	20.1 ± 0.8	18.3 ± 0.7	0.50	0.20
Alcohol (g)	12.1 ± 2.1	11.0 ± 2.1	5.8 ± 1.7	0.70	0.08
Calcium (mg)	1226 ± 44	1300 ± 53	677 ± 41	0.60	< 0.001

^a^All values are means ± SEMs, *n* = 79 because 6 (subjects had dietary records considered nonsufficient and were therefore removed from the model). Data were assessed by using 3-d weighted dietary record estimated using Dankost 3000 dietary assessment software (Dankost). Statistical differences were based on linear mixed models with values for average daily consumption at week 0 for all variables included as covariates. Pairwise comparisons were based on post hoc t- test with *p*-values adjusted for multiplicity. Abbreviations: *CHO* carbohydrate control, *RED* reduced-fat cheese, *REG* regular-fat cheese

## Discussion

In the present study, we compared the effects of consuming regular-fat cheese, reduced-fat cheese and carbohydrate-rich foods for 12 wks on lipoprotein particle size distribution in a population with ≥2 metabolic syndrome (MetS) risk factors. Our results demonstrated that long-term consumption of regular-fat cheese did not modify the distribution of LDL subclasses differently compared with reduced-fat cheese. The lack of differences in LDL particle size between the two cheese groups were unexpected, as the saturated fat intake in the regular-fat cheese group was significantly higher than in the reduced-fat cheese group (33.3 g/d vs. 27.2 g/d). However, it can be speculated that the differences in saturated fat were insufficient to elicit a significant shift towards larger LDL particles in the regular-fat cheese group, as overall fat intake did not differ between the two groups. Furthermore, it cannot be ruled out that the effect of fat in cheese on LDL particle size might be modulated by constituents in the cheese matrix. The high content of calcium in cheese has been suggested to modulate the effect of saturated fat on LDL-C concentrations by increasing fecal fat excretion. Measurements of fecal fat and energy excretion in this study could have added to the mechanistic understanding of the observed findings. Our finding is in accordance with a recently published study showing that despite a 6% higher saturated fat content in a high-fat dairy DASH diet compared with a low-fat dairy DASH diet, no differences in LDL cholesterol or any of the LDL subclasses was observed in a population of both men and women [25]. In the present study, however, significant gender-specific differences in regard to LDL subfractions were observed. In men, total LDL particle number was significantly reduced following the REG diet compared with the RED diet. The decrease in total LDL particle number was primarily due to a ~ 6% lower proportion of midsize LDL particles, while the proportion of smaller LDL particles only tended to be reduced (*P* = 0.06). In contrast to the results obtained in men, the REG diet only tended to increase total LDL-P compared with the RED diet in women (*P* = 0.08). Our findings indicate that middle-aged men might have a more favorable lipoprotein response to regular-fat cheese compared to reduced-fat cheese than middle-aged/postmenopausal women, although this needs to be verified in further studies with larger sample size. However, LDL particle size distribution depends on other factors than gender and genetics alone, including, hormonal status (at menopause, LDL particle size distribution shift towards sdLDL) [26], overall lifestyle, and dietary habits. These factors can be difficult to adjust for in a study with “free living” individuals. Thus, it cannot be ruled out that the gender differences in response to the test foods in the current study have been influenced by these additional factors.

The saturated fat in regular-fat cheese did not result in larger LDL particles compared to the carbohydrate control, despite the significantly higher intake of saturated fat (33.3 g/d vs. 19.0 g/d). This was unexpected, as individuals who consume diets high in saturated fat appear to have larger LDL particles compared with diets high in carbohydrates [9, 10]. However, most of these studies were conducted in normolipidemic men and not in a population of men and women with risk factors for the MetS as in the current study. Also, these studies are often carried out with carbohydrate restriction in the high-fat groups. However, the fact that subjects in this study were not carbohydrate-restricted in the regular-fat cheese group might explain the lack of difference.

The present study confirmed previous observations that diets with higher fat content increases HDL-C compared to carbohydrate-rich diets [27]. Overall, the concentration of total HDL-C was significantly higher for the regular-fat cheese diet compared with the carbohydrate-rich diet. The increase in total HDL-C was due to a higher concentration of cholesterol carried in the smaller HDL subclasses. Interestingly, a gender sub analysis revealed that these differences in HDL-C were only true for women. However, as this study did not have results on HDL particle size distribution, the study cannot confirm if changes in HDL-C concentrations in women were due to changes in particle number or HDL composition. In men, no significant differences were observed in HDL-C after the REG diet compared to the CHO diet. But, HDL-C in the larger HDL particles tended to be higher following the REG diet compared with the RED diet (*P* = 0.09). Whereas small, dense LDL particles have been associated with higher incidence of CVD and diabetes, the relation between HDL subfractions and CVD outcomes are more controversial. Large HDL particles have been suggested to be more cardio protective than small HDL particles [28–30], although not consistently [31, 32].

Despite the relatively large amount of intervention studies investigating the effects of cheese intake on the standard lipid profile compared with other dairy products [33–37], evidence regarding the effect of cheese on LDL particle size and lipoprotein subclasses remains scarce. In fact, the present study is to our knowledge the first randomized controlled feeding trial to evaluate the cardiometabolic effects of cheese intake beyond the standard lipid profile (TC, LDL-C and HDL-C). However, a few studies have investigated the impact of other dairy foods on LDL particle size [38–41]. In 35 healthy men and women (> 27 kg/m2) the impact on LDL particle size of low-fat dairy (500 mL low-fat milk and 150 g low-fat yogurt) was compared with carbohydrate-rich foods (600 mL fruit juice and 3 fruit biscuits). The low-fat dairy did not alter LDL or HDL particle number compared with the carbohydrate diet [39]. In 14 normolipidemic men and women, the intake of 500 ml/d nonfat milk, but not whole-fat milk, was associated with larger LDL particles during a 2 wk. intervention [40]. In a 6-wk randomized, crossover study, in which 27 postmenstrual women consumed a diet with 3.2 servings/day of 2% fat milk or a milk and dairy free diet, no differences was observed on LDL particle size [38]. In healthy men, consumption of full-fat dairy (milk, butter, and cheese) and dairy products enriched with naturally derived *trans* fatty acids was not associated with changes in LDL particle size [41]. Lastly, consumption of high-fat dairy diet for 3 wk. did not alter LDL particle size, whereas consumption of low-fat dairy reduced LDL particle size compared with a control diet [25]. The results from these studies suggest that the effect of dairy on LDL particle size differs in regard to type of dairy product consumed, metabolic status of the population, and gender. Thus, more studies are needed to fully elucidate the impact of cheese and other dairy products on LDL particle size distribution.

The strength of the current study was its long duration, as it increased our ability to detect changes in lipoprotein profile over a longer time span. The inclusion of a 2-wk run-in period with dietary restrictions (non-dairy restriction) is also strength as it allowed the subjects to physically adapt to the dietary changes and thereby help adjust for prior dietary habits. The fact that subjects maintained stable body weight throughout the intervention is an advantage, which (together with the statistical adjustment for individual changes in body weight) supports, that our results were independent of changes in body weight. Limitations of the present study include the fact that no power calculation was used; thus a potential risk of reporting false negatives. Compliance in this study was based on self-reporting, thus a potential risk of response bias is present. Also, the lack of biomarkers for assessment of dietary compliance is a limitation in this study. Lastly, because of the small sample size, especially in men, generalizability of the study results is limited and further research is therefore needed to determine the effect of regular-fat cheese on LDL particle size distribution.

## Conclusions

In conclusion, regular-fat cheese did not alter overall LDL particle size distribution compared to reduced-fat cheese after a 12 wk. intervention in subjects with MetS. However, our results suggest that lipoprotein response to cheese intake is gender-specific. This warrants further investigation.

## Abbreviations

Apo: Apolipoprotein
CHO: Carbohydrate control
CVD: Cardiovascular disease
HDL-C: High density lipoprotein cholesterol
IDL-C: Intermediate density lipoprotein cholesterol
IDL-P: Intermediate density lipoprotein particle number
LDL-C: Low density lipoprotein cholesterol
LDL-P: Low density lipoprotein particle number
RED: Reduced-fat cheese
REG: Regular-fat cheese
VLDL-C: Very low density lipoprotein cholesterol
VLDL-P: Very low density lipoprotein particle number
